# Unveiling the Realm of Denture Fabrication: Revitalizing Aesthetics and Optimizing Efficiency for Geriatric Patients

**DOI:** 10.7759/cureus.50392

**Published:** 2023-12-12

**Authors:** Ankita Pathak, Mithilesh M Dhamande, Seema Sathe, Smruti Gujjelwar, Sheetal R Khubchandani, Dhanashree A Minase

**Affiliations:** 1 Prosthodontics, Sharad Pawar Dental College and Hospital, Datta Meghe Institute of Higher Education and Research, Wardha, IND

**Keywords:** cheek plumpers, neutral zone, severely resorbed mandibular ridge, flabby tissue, complete denture

## Abstract

Disfigurement of the face has a significant impact not only on the appearance and function but also on the psychological well-being of an individual. Due to tooth loss, various psychological problems can occur. Dentures can help patients regain a sense of normalcy and also boost their confidence to live in society. Complete denture restores phonetics, esthetics, and mastication. A 63-year-old male patient reported to the Department of Prosthodontics with the chief complaint of total loss of teeth and wanted to get treated for the same. The purpose of this article is to describe changes in impression techniques, dental material, methodologies in teeth setting, and novel approaches to enhance the esthetics to optimize the results in a final complete denture. To reestablish the patient’s masticatory, apparatus steps of complete denture fabrication were revisited accompanied by the modern application of innovative techniques with the base of historical concept.

## Introduction

Edentulism is a long-term disability that makes it challenging for edentulous individuals to perform daily tasks, including eating, speaking, and interacting with others [1]. The loss of facial muscular support, biting leverage, and decreased masticatory efficiency are all physical effects of tooth loss [2]. To achieve a favorable outcome for denture therapy, the impression technique should be based on the current status of the mandible's basal tissue support [3]. In this article, the neutral zone paradigm was employed for impression recording to create successful mandibular complete dentures [4,5].

According to Kelly, extensive maxillary and mandibular ridge resorption and alveolar bone loss can result in the "flabby ridge," a mobile piece of tissue mainly seen on rugae and the tuberosity region in the maxilla, as well as the anterior crest of the ridge and retromolar area in the mandibular denture [6]. This type of soft tissue is mostly present in denture wearers in the long run, leading to discomfort and ill-fitting dentures [7-9].

In today's image-conscious society, dentures restore a natural appearance, encouraging patients' confidence and facilitating easier social interactions. Thus, facial aesthetics are vital [10,11]. The application of cheek plumpers can provide support to oral musculature in geriatrics. This clinical report describes a less invasive method for addressing all the mentioned problems, enhancing not only aesthetics but also optimizing the results of wearing complete dentures in long-term denture wearers.

## Case presentation

**Figure 1:**
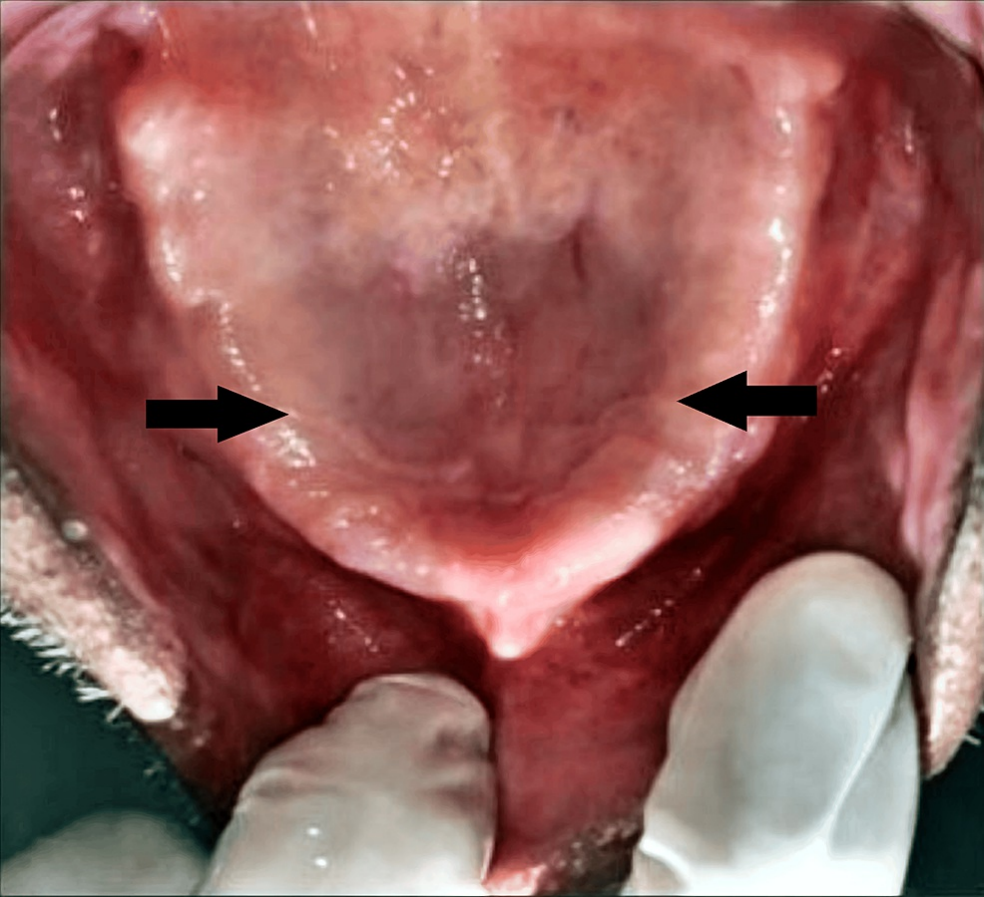
Edentulous maxillary arch Image credit: Ankita Pathak

**Figure 2:**
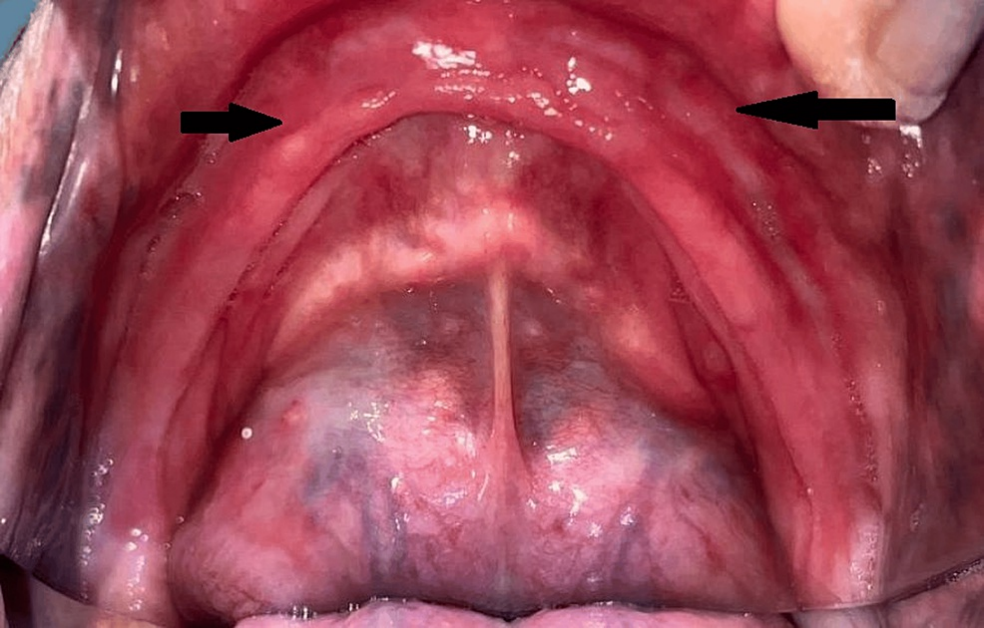
Severely resorbed mandibular arch Image credit: Ankita Pathak

A 63-year-old male patient reported to the Department of Prosthodontics with the chief complaint of ill-fitting dentures. The patient had been using dentures for 20 years and had gone through five sets, none of which were satisfactory. The major concerns were restoring the lost masticatory apparatus and addressing the ill-fitting of dentures caused by flabby tissue. Flabby tissue in the anterior maxillary region and extensive resorption in the mandibular ridge were noted, as shown in Figures 1, 2.

A thorough intraoral examination revealed unsupported oro-facial muscles and sunken cheeks. The treatment plan was formulated with consideration for the patient's mindset. To address the issue of sunken cheeks, a magnetically retained detachable cheek plumper was designed to complement the dentures.

The preliminary impressions for the maxillary arch were made with irreversible hydrocolloid impression material and the severely resorbed mandibular ridge was recorded with an admix technique. Final impression techniques were refined, incorporating different methods for specific challenges. Hobkirk's technique recorded the flabby maxillary arch, while the severely resorbed mandible was recorded with light-body polyvinyl siloxane (PVS) over an admixed technique. These techniques were employed in addition to routine conventional impression techniques.

A high-viscosity PVS putty material was manipulated and adapted onto a modified record base with orthodontic wire, which was then placed in the patient's mouth. The patient was instructed to perform normal mandibular actions, such as mastication, lip-sucking, and vowel pronouncing, to aid in shaping the neutral zone area.

**Figure 3:**
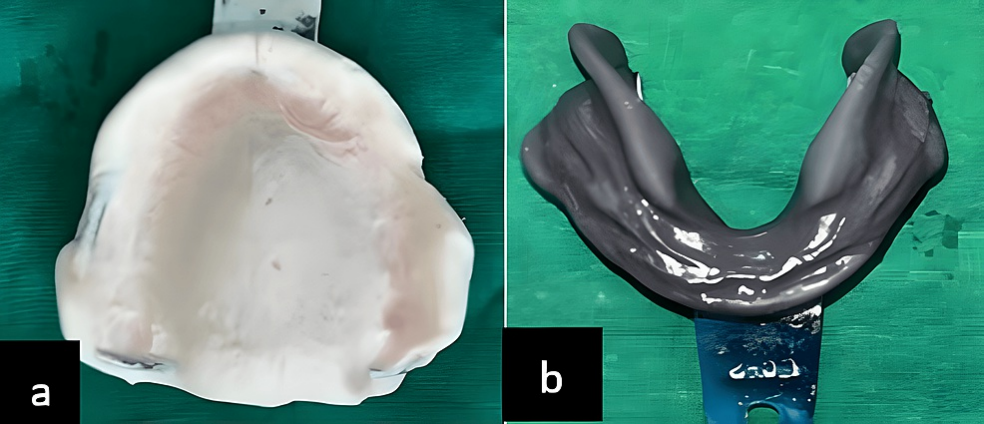
(a) Preliminary impression of maxilla and (b) preliminary impression of mandible Image credit: Ankita Pathak

Following the diagnosis and evaluation of soft tissue, maxillary, and mandibular ridges, a preliminary impression was made. The maxillary primary impression utilized an irreversible hydrocolloid impression material (Zhermack Dust-free Thixotropic Tropicalgin, Zhermack SpA, Badia Polesine [RO], Italy), while the mandibular primary impression was made with impression compound and green stick (DPI - Pinnacle Impression Compound and Tracing Sticks, The Bombay Burmah Trading Corporation, Ltd., Mumbai, India) using the admix technique, in which impression compound and green stick were taken in 3:7 proportion, kneaded, and adapted on the stock tray to make the impression, as depicted in Figure 3.

Special trays were fabricated over the primary cast, trimmed 2mm short to provide space for a green stick border molding. Modifications were made to the final impression techniques to capture the challenges presented by a severely resorbed mandible and flabby tissue in the mandible.

The all-green technique was employed for the mandibular resorbed ridge, over which a wash impression was recorded using light-body PVS. In contrast, the maxillary arch was recorded using Hobkirk's technique, with flabby tissue recorded using light viscosity, and the remaining region recorded with a regular viscosity PVS impression material [1,2,5].

The term "flabby ridge" pertains to the pliable and mobile tissue found in both the maxillary and mandibular ridges, with a particular emphasis on the anterior region of the maxilla in long-term denture wearers. During the impression-making process, forces applied may cause distortion in these mobile tissues. Failure to appropriately manage this mobility can lead to displacement of the denture due to masticatory forces, resulting in compromised retention, support, and stability.

**Figure 4:**
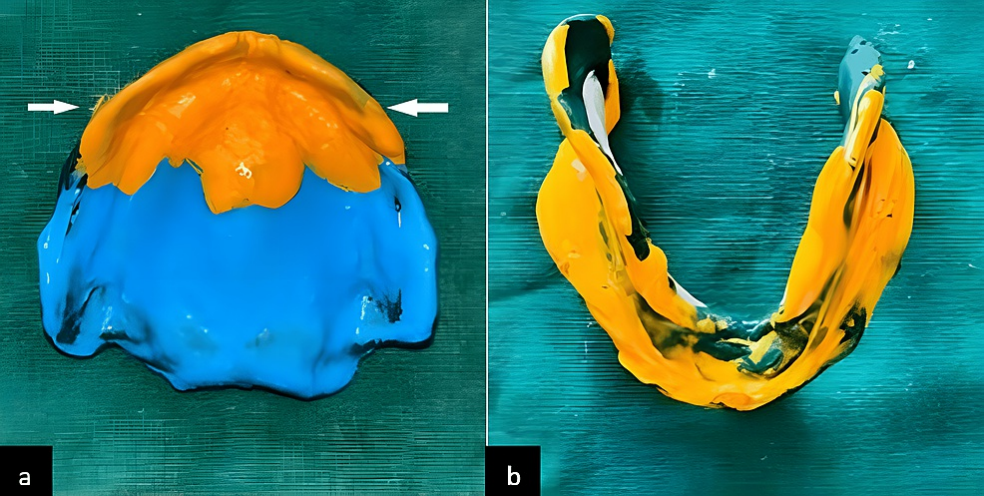
(a) Final impression of the maxilla and (b) final impression of the mandible Image credit: Ankita Pathak

To address the challenge posed by displaceable denture-bearing tissue, various approaches can be considered. Surgical interventions, specialized impression techniques, and ensuring a balanced distribution of occlusal pressures are potential strategies. Additionally, implant therapy represents another viable option for managing flabby ridges. By carefully addressing these factors, clinicians can mitigate the adverse effects of mobile tissue distortion and enhance the overall performance of dentures for individuals with flabby ridges. The final impression techniques are illustrated in Figure 4.

**Figure 5:**
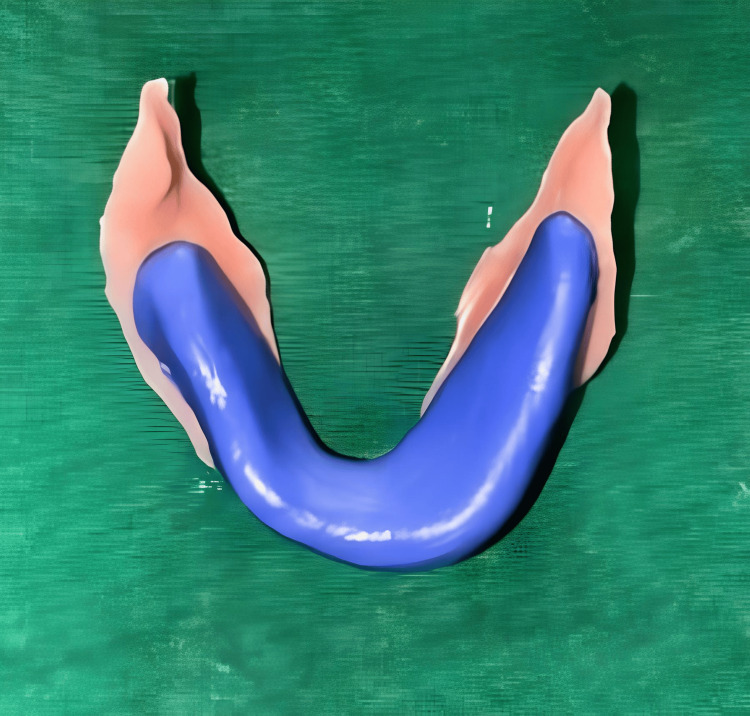
Recorded neutral zone Image credit: Ankita Pathak

For recording the neutral zone, a mandibular record base was made with an orthodontic wire was adapted to retain putty, and kneaded putty (Chromaclone™ PVS Putty - Ultradent, Germany) was adapted over it. Subsequently, the patient was instructed to perform functional movements and actions, including swallowing, puckering lips, and pronouncing various vowels such as "oo" and "me." Following these activities, the neutral zone base plate was removed from the patient's mouth. The recorded neutral zone is depicted in Figure 5.

**Figure 6:**
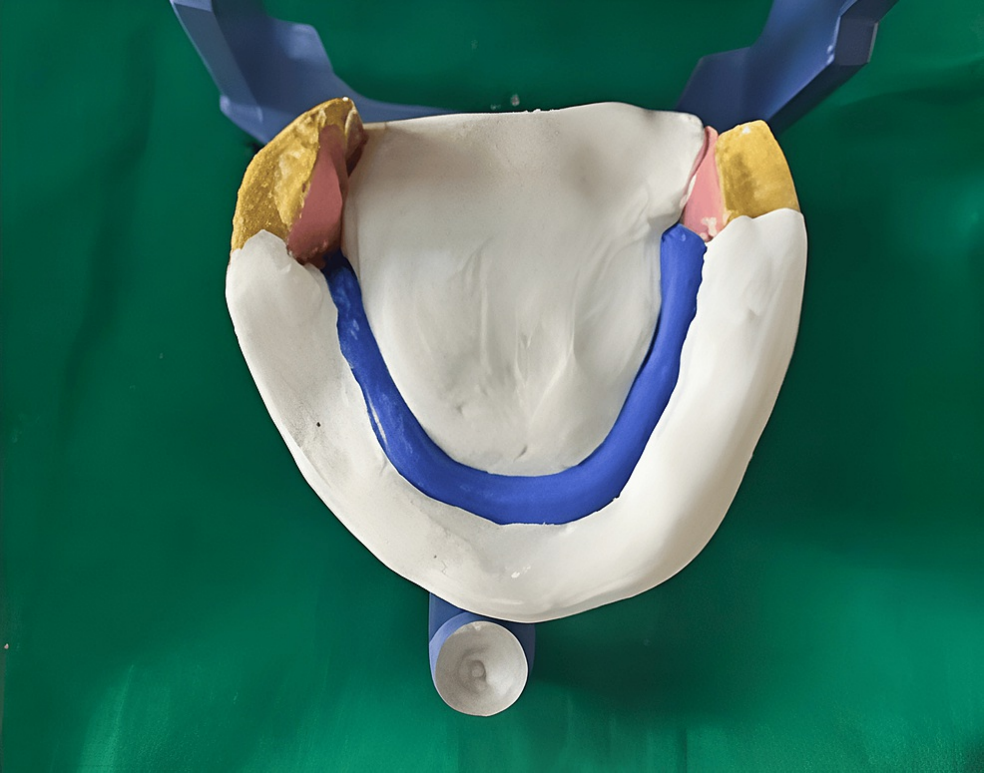
Neutral zone index Image credit: Ankita Pathak

The occlusal vertical dimension and the centric relation using the swallowing method were transferred onto the articulator. Subsequently, the mandibular base plate was removed, and the recorded neutral zone denture base was positioned on the articulator. A petroleum jelly was applied on the cast, type two dental stone used to make plaster index. The neutral zone space was retained with a plaster index, as illustrated in Figure 6.

Subsequently, the putty was removed from the denture base, and molten modeling wax (Pyrax Dental Modelling Wax, Germany) conformed to the shape of the neutral zone space. This new space was then filled by pouring wax between the index. Teeth setting was performed in this modified space.

Taking into consideration the patient's complaint about difficulty in mastication, anatomic teeth were chosen to address masticatory challenges. The teeth arrangement, wax-up, and carving for aesthetics were employed for the try-in. Despite examining the patient's aesthetics, phonetics, and mastication, dissatisfaction with appearance persisted due to sunken cheeks.

To address drooping cheekbones and enhance facial aesthetics, a longitudinal bulb of softened modeling wax was placed on the buccal flange area of the maxillary denture. This was secured to the maxillary region using magnets during the try-in stage. Consequently, magnetic removable cheek plumpers were created to meet the patient's needs. Detachable bulbs were placed on both sides of the buccal flange area and molded separately with the entire dentures. These were then repaired, finished, and polished.

**Figure 7:**
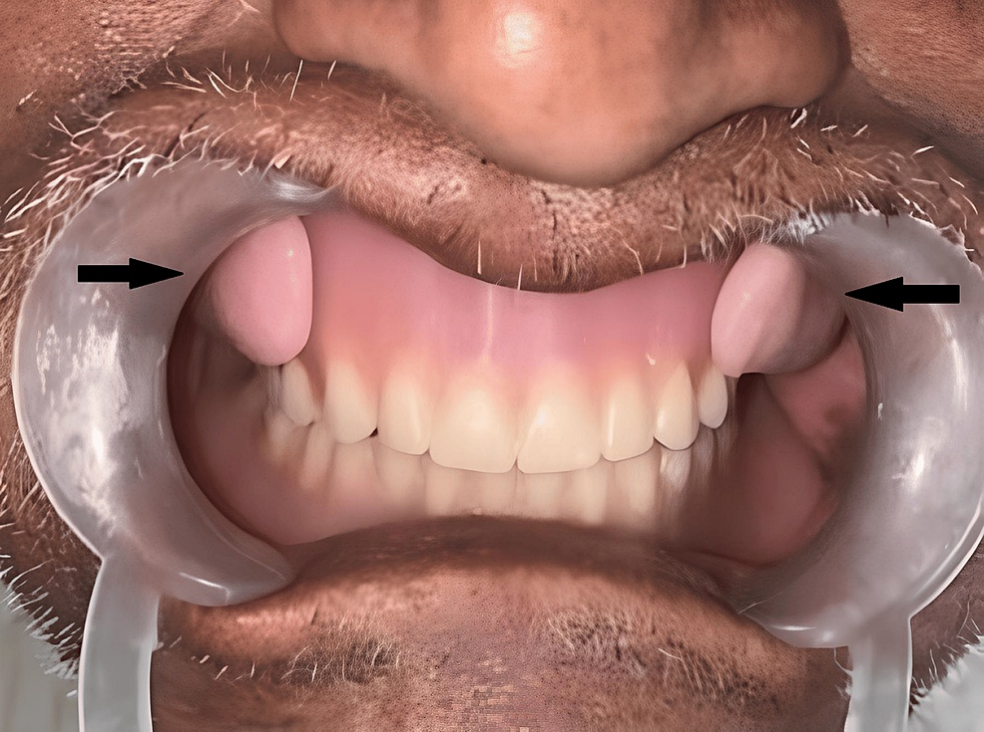
Complete denture insertion with magnet-retained cheek plumpers Image credit: Ankita Pathak

All essential denture properties, including retention, stability, and support, were thoroughly examined. After occlusion verification, selective grinding was performed, reducing upper buccal cusps and lower lingual cusps. Occlusion was once again verified, and the patient found comfort with the new set of complete dentures, as depicted in Figure 7.

The patient was given a demonstration on the proper wearing of dentures and cheek plumpers. Post-insertion instructions were provided to the patient, and to ensure retention, comfort, and function, three recall visits were scheduled after three days, one week, and 15 days successively.

## Discussion

In the future, tooth loss will persist as an ongoing concern, necessitating a prosthetic approach for replacement. Complete dentures, to a certain extent, restore both aesthetics and function. While the majority of denture wearers adapt to their disability, others may face significant psychological and functional challenges [12]. Additionally, any surgical procedure carries inherent risks of complications. To address these issues, functional impression techniques have been developed to overcome challenges and fabricate successful prostheses. Anatomic teeth are utilized for a balanced occlusion scheme to enhance masticatory efficiency [13]. Instead of resorting to surgical interventions with potential extensive scarring, broadening the fundamental principles of fabricating complete dentures can yield excellent results [14].

Even the most aesthetically pleasing dentures are rendered ineffective if they shift during speech and social interaction [15]. Following the diagnostic and preparation phases of counseling, impression visits allow the dentist to confirm the diagnosis of oral diseases while also gauging the expected degree of patient compliance with the treatment. Impression is an imprint created by the pressure of one thing upon or into the surface of another. The dentist should remember that impressions are made rather than taken [16]. The edentulous oral cavity must be restored to as healthy as possible before creating new dentures. Failing to recognize this requirement and incorporating essential procedures in the treatment plan hinders a satisfactory prognosis [17-21].

Applying unique and different techniques at each step, rather than routine procedures, will optimize the results of complete denture fabrication.

## Conclusions

In summary, dentistry faces the ongoing challenge of addressing tooth loss, necessitating effective prosthetic solutions. Complete dentures, though providing a degree of aesthetic and functional restoration, present varying degrees of adaptation among wearers. The achievement of well-supported cheeks, sufficient retention, and exceptional stability was optimized in the complete denture delivered to the patient through various impression techniques, leading to a more youthful appearance for the individual. Impression visits play a vital role in confirming diagnoses and assessing patient compliance. Viewing impressions as active creations rather than passive imprints underscores their importance in fabrication. Investing in a robust primary impression is essential, and restoring edentulous mouths to a healthy state is foundational for successful denture outcomes. Adopting unique techniques at each step of the fabrication process for complete dentures can lead to optimized results compared to strictly following routine procedures.

## References

[1] Bansod AV, Pisulkar SG, Dahihandekar C (2022). Enhancing esthetics in a complete denture patient: optimizing results with different impression techniques. Cureus.

[2] Yadav M, Sardar C, Mistry G, Bachhav M (2021). Flabby tissue-revisited: case report. Int J Appl Dent Sci.

[3] Raj KS, Suganya S, Malode G (2021). Prosthodontic rehabilitation of compromised upper and lower residual alveolar ridges: a case report. IP Ann Prosthodont Restor Dent.

[4] Wee AG, Cwynar RB, Cheng AC (2000). Utilization of the neutral zone technique for a maxillofacial patient. J Prosthodont.

[5] Bansal R, Kumar M, Garg R, Saini R, Kaushala S (2014). Prosthodontic rehabilitation of patient with flabby ridges with different impression techniques. Indian J Dent.

[6] Kelly E (1972). Changes caused by a mandibular removable partial denture opposing a maxillary complete denture. J Prosthet Dent.

[7] Al-Jammali ZM, Saad M (2020). Prosthodontics clinical cases (patients with decreased inter-arch distance-case report). IJMRA.

[8] Marin DO, Leite AR, de Oliveira Junior NM, Compagnoni MA, Pero AC, Arioli Filho JN (2015). Reestablishment of occlusal vertical dimension in complete denture wearing in two stages. Case Rep Dent.

[9] Matsuda R, Yoneyama Y, Morokuma M, Ohkubo C (2014). Influence of vertical dimension of occlusion changes on the electroencephalograms of complete denture wearers. J Prosthodont Res.

[10] Hansen CA (1985). Diagnostically restoring a reduced occlusal vertical dimension without permanently altering the existing dentures. J Prosthet Dent.

[11] Silverman MM (1953). The speaking method in measuring vertical dimension. J Prosthet Dent.

[12] Tallgren A, Lang BR, Walker GF, Ash MM Jr (1980). Roentgen cephalometric analysis of ridge resorption and changes in jaw and occlusal relationships in immediate complete denture wearers. J Oral Rehabil.

[13] Kazmi SM, Qureshi S, Iqbal Z (2013). Window’s impression technique for anterior fibrous maxillary ridges. J Dow Univ Health Sci.

[14] Watt DM, MacGregor AR (1976). Designing Complete Dentures. https://www.semanticscholar.org/paper/Designing-Complete-Dentures-Watt-MacGregor/bd0f8fb4954466d429197509a6964017d25789c3.

[15] Besford JN, Sutton AF (2018). Aesthetic possibilities in removable prosthodontics. Part 2: start with the face not the teeth when rehearsing lip support and tooth positions.. BDJ.

[16] McCord JF, Grant AA (2000). Impression making. Br Dent J.

[17] Bergman B, Carlsson G (1985). Clinical long-term study of complete denture wearers. J Prosthet Dent.

[18] Martone AL (1963). Clinical applications of concepts of functional anatomy and speech science to complete denture prosthodontics: Part VII. Recording phases. J Prosthet Dent.

[19] Boos R. H (1957). Complete denture technique, including preparation and conditioning. Clin N America.

[20] Edwards LF, Boucher CO (1942). Anatomy of the mouth in relation to complete dentures. J Am Dent Assoc.

[21] Pilon JF, Olthof A, van de Poel AC (1985). The construction of a lower denture from the form of the neutral zone in the edentulous mouth (Article in Dutch). Ned Tijdschr Tandheelkd.

